# AMRColab – a user-friendly antimicrobial resistance detection and visualization tool

**DOI:** 10.1099/mgen.0.001308

**Published:** 2024-10-21

**Authors:** Su Datt Lam, Sabrina Di Gregorio, Mia Yang Ang, Emma Griffiths, Tengku Zetty Maztura Tengku Jamaluddin, Sheila Nathan, Hui-min Neoh

**Affiliations:** 1 Department of Applied Physics, Faculty of Science and Technology, Universiti Kebangsaan Malaysia, Bangi, Malaysia; 2 Center for Global Health Research (CGHR), Saveetha Medical College, Saveetha Institute of Medical and Technical Sciences (SIMATS), Saveetha University, Chennai, India; 3 Instituto de Investigaciones en Bacteriología y Virología Molecular (IBaViM), Facultad de Farmacia y Bioquímica, Universidad de Buenos Aires, Buenos Aires, Argentina; 4 Consejo Nacional de Investigaciones Científicas y Técnicas (CONICET), Buenos Aires, Argentina; 5 Department of Diagnostic & Allied Health Science, Faculty of Health & Life Sciences, Management & Science University, Shah Alam, Selangor, Malaysia; 6 Centre for Infectious Disease Genomics and One Health, Simon Fraser University, Vancouver, British Columbia, Canada; 7 Department of Medical Microbiology, Faculty of Medicine and Health Sciences, Universiti Putra Malaysia, Serdang, Malaysia; 8 Department of Biological Sciences & Biotechnology, Faculty of Science & Technology, Universiti Kebangsaan Malaysia, Bangi, Malaysia; 9 UKM Medical Molecular Biology Institute (UMBI), Universiti Kebangsaan Malaysia, Kuala Lumpur, Malaysia

**Keywords:** antimicrobial resistance (AMR), bioinformatics, genomic surveillance, Google Colaboratory

## Abstract

Antimicrobial resistance (AMR) poses a significant threat to global public health, with the potential to cause millions of deaths annually by 2050. Effective surveillance of AMR pathogens is crucial for monitoring and predicting their behaviour in response to antibiotics. However, many public health professionals lack the necessary bioinformatics skills and resources to analyse pathogen genomes effectively. To address this challenge, we developed AMRColab, an open-access bioinformatics analysis suite hosted on Google Colaboratory. AMRColab enables users with limited or no bioinformatics training to detect and visualize AMR determinants in pathogen genomes using a ‘plug-and-play’ approach. The platform integrates established bioinformatics tools such as AMRFinderPlus and hAMRonization, allowing users to analyse, compare and visualize trends in AMR pathogens easily. A trial run using methicillin-resistant *Staphylococcus aureus* (MRSA) strains demonstrated AMRColab’s effectiveness in identifying AMR determinants and facilitating comparative analysis across strains. A workshop was conducted and feedback from participants indicated high confidence in using AMRColab and a willingness to incorporate it into their research. AMRColab’s user-friendly interface and modular design make it accessible to a diverse audience, including medical laboratory technologists, medical doctors and public health scientists, regardless of their bioinformatics expertise. Future improvements to AMRColab will include enhanced visualization tools, multilingual support and the establishment of an online community platform. AMRColab represents a significant step towards democratizing AMR surveillance and empowering public health professionals to combat AMR effectively.

**Impact Statement**

The existing antimicrobial resistance (AMR) of clinically important pathogens is a global problem. To combat, manage and maintain proper records of AMR at the country level, it is important to conduct routine surveillance to predict pathogen behaviour in their response towards antibiotics. One effective approach is whole genome sequencing (WGS), but this requires laboratory staff who are competent in bioinformatics and the use of appropriate tools to consolidate and compare AMR pathogen sequences across laboratories and public health facilities. However, challenges faced by a number of countries include lack of equipment for WGS while bioinformatics analyses of pathogen genomes pose a test for many public health professionals, as genomic analysis is a relatively new field for these professionals. To overcome this issue, we developed ‘AMRColab’, a Google Colaboratory (Colab) prototype to allow users with limited or no bioinformatics skills to detect and visualize AMR determinants on any computing device, even in low-resource settings. This would allow timely surveillance of AMR pathogens by local health authorities, leading towards prevention of AMR transmission.

## Data Summary

Genome sequences of six methicillin-resistant *Staphylococcus aureus* (MRSA) strains isolated from Malaysia [accession numbers: PPUKM-261-2009 (AMRB00000000), PPUKM-377-2009 (AMRD00000000), PPUKM-775-2009 (AMRE00000000) and M106_2017 (JALLIT000000000)] and Argentina [SRA numbers: 2028P (SRS5588740) and CF39 (SRS4380564)] are deposited in the National Center for Biotechnology Information (NCBI) Sequence Reads Archive (SRA) public database. The course manual is available at https://zenodo.org/records/6438173.

## Introduction

Antimicrobial resistance (AMR) is a pressing global public health concern [1], with many pathogenic microbes having gained resistance towards existing drugs, while the development of new antimicrobials has slowed down. The World Health Organization (WHO) estimates that drug-resistant bacterial infections will cause up to 10 million deaths worldwide by 2050 [2]. Unmanaged AMR could shave up to 3.8% off global GDP per year by 2050, potentially driving 28 million people into poverty, as predicted by the World Bank in 2017 [3].

To effectively combat and manage AMR, surveillance of AMR pathogens via whole genome sequencing (WGS) is crucial to monitor and predict pathogen behaviour in their response towards antibiotics. Various bioinformatics tools have been developed to assemble raw genome sequence reads and to identify AMR determinants (genes and point mutations) in pathogen genomes [4]. Following that, specialized bioinformatics tools, such as hAMRonization [5], are utilized to standardize the output of different AMR determinant detection software and facilitate the comparison of AMR pathogen genome sequences across laboratories and countries. In addition, various machine learning algorithms have been proposed towards identifying AMR [6,7].

Despite the availability of different software and algorithms, bioinformatics analyses of pathogen genomes pose a challenge for many public health professionals [8,9]. Most lack the necessary Linux-based machines or coding skills to use currently available bioinformatics tools effectively. The cost of genome sequencing equipment is getting cheaper (e.g. the MinION nanopore sequencer now costs USD1000), which has enabled some low-resource laboratories to procure sequencers for AMR surveillance. Nonetheless, these laboratories still struggle with data analysis. As a result, numerous hospital laboratories and public health organizations resort to commercial bioinformatics service providers to analyse AMR trends. This practice is costly and neither ideal nor sustainable for long-term AMR management, especially for low-resource settings.

To overcome the bioinformatics barrier in managing AMR, we developed ‘AMRColab’, a Google Colaboratory (Colab) (a free Google Notebook that allows bioinformatics code to be launched from a web browser [10]) prototype, that enables users with limited or no bioinformatics skills to detect and visualize AMR determinants on any computing device, even in low-resource settings. With ‘AMRColab’, users without bioinformatics training will be able to easily analyse, compare and visualize trends of circulating AMR pathogen genomes, allowing timely surveillance of these pathogens by local health authorities, leading towards prevention of AMR transmission.

## Methods

### AMRColab development

AMRColab was developed via plugging three modules in a Google Colab, namely AMRFinderPlus [11] developed by the National Center for Biotechnology Information (NCBI) (https://github.com/ncbi/amr), hAMRonization [5] developed by the Public Health Alliance for Genomic Epidemiology (https://github.com/pha4ge/hAMRonization), and a visualization module containing Pathogenwatch [12] (https://pathogen.watch/) and Microreact [13] (https://microreact.org/), both web-based applications developed by the Centre for Genomic Pathogen Surveillance.

Colabs are Jupyter Notebooks accessible online by Google and require no unique computer setup. Via Colab notebooks, applications that require huge processing power only achievable via GPUs and TPUs can be freely accessed via the web by computers in under-resourced laboratories, as long as there is internet access.

After creation of a Google Colaboratory account, AMRFinderPlus was first plugged into the notebook. We included another python code to produce a simplified text file containing only determinants associated with AMR. AMRFinderPlus replaced its forerunner AMRFinder. It is a widely used bioinformatics tool to identify AMR genes and AMR-associated mutations in bacterial genomes. The tool also detects genes associated with metal, heat, acid, biocide and stress resistance, as well as virulence genes.

Following this, the second module, hAMRonization [5], a bioinformatics tool to allow a standardized comparison of AMR determinants from different bacterial genomes, was plugged into the Colab. hAMRonization consists of a python package and requires command-line knowledge to utilize it. The tool works to combine outputs of different AMR determinant detection tools (AMRFinderPlus in the case of AMRColab) into a single spreadsheet.

To enable visualization of AMR determinants, hAMRonization output was converted into a Microreact-ready .csv file, and instructions on how to use Pathogenwatch and Microreact were subsequently embedded into the Colab as the third module. We used the ability of Pathogenwatch to reconstruct a phylogenetic tree based on a core genome distance for a curated set of species (detailed on the website https://pathogen.watch/) and the ability of Microreact to display and integrate results. Addition of these plug-ins into AMRColab allows outputs from hAMRonization to be presented graphically in the form of phylogenetic/genomic trees with accompanying geographical and temporal metadata of strains being investigated.

Upon completion of the Colab, running all modules from the Colab will enable users to upload genomes of tested bacteria, identify AMR determinants in these genomes, and compare and visualize AMR determinant content between bacteria (even from different laboratories), only by following the prompts from the Colab; no bioinformatics skills are required to run the analysis. AMRColab also allows bulk processing of multiple genomes in a single analysis. A manual describing AMRColab has been developed and is available at: https://zenodo.org/records/6438173.

### AMRColab testing

Genome sequences of six methicillin-resistant *Staphylococcus aureus* (MRSA) strains isolated from Malaysia (PPUKM-261-2009, PPUKM-377-2009, PPUKM-775-2009 and M106_2017 [14,15]) and Argentina (2028P and CF39 [16,17]) were used as the test set for the Colab. Subsequently, these genomes were later used as examples during introductory workshops of the Colab.

### Workshop

Two Zoom-based online workshops were organized on 13 April 2022 and 12 April 2023 to introduce AMRColab and its utility in AMR management. Targeted participants were those from local universities and research institutes who were undertaking WGS on AMR bacteria and comparing AMR determinants between bacterial strains obtained from different laboratories. We also invited researchers from public health laboratories who were generating reports on surveillance of AMR bacteria.

The programme of the two workshops consisted of a lecture on AMR, AMR databases and AMR gene prediction tools, a lecture to introduce AMRColab and a (virtual) hands-on session in three different Zoom breakout rooms. The second workshop also included a lecture to introduce phylogenetic tree reconstruction and interpretation. The concluding workshop activity was the presentation of results generated from AMRColab by representative workshop participants. Participants of both workshops were requested to complete a Google Form to obtain their feedback on AMRColab. A total of three and eight questions were posed within the 2022 and 2023 workshop feedback forms, respectively (Table 1). In March 2024, all participants were requested to complete a new feedback form to gauge post-workshop utilization of AMRColab.

**Table 1.** Questions on the feedback form for the AMRColab workshop participants

Workshop	Questions
AMRColab 2022	Do you feel confident to use the AMRColab pipeline after the workshop?
	Will a standardized AMR report (as produced by AMRColab) be useful in your work settings?
	Will you be interested in attending an advanced workshop on AMRColab?
AMRColab 2023	Will a standardized AMR report (as produced by AMRColab) be useful in your work settings?
	Do you feel confident to use AMRColab after the workshop?
	Will you be using AMRColab in your research?
	Will the output of AMRColab be useful in your surveillance work?
	How likely is it that you will be recommending AMRColab to a colleague?
	Will you be interested in attending an advanced workshop on AMRColab?
	How easy is it to follow the user manual (rank 1–5, 5 is the highest score)?
	AMRColab is easy to use (rank 1–5, 5 is the highest score)
Post-workshop utilization survey	Have you managed to use AMRColab in your work?
	Do you find AMRColab helpful in your work on AMR?
	What are your expectations for an online, end-to-end bioinformatics solution for surveilling AMR genomes?

## Results and discussion

AMRColab was developed using the Google Colaboratory platform. A Colab allows users without bioinformatics knowledge to execute programs written in Python codes in a ‘plug-and-play’ manner. With the development of AMRColab, the AMRFinderPlus and hAMRonization bioinformatics tools used for AMR analysis can be utilized by any medical laboratory technologist, medical doctor and public health scientist via a few clicks of a mouse attached to a personal computer with stable internet connection. AMR comparison results from the analysis are then viewable using the Microreact plug-in, where the results could be shared quickly and discussed among collaborators. The Colab is stored in Google Drive and is currently free of charge to be used at any location.

### AMRColab interface

**Fig. 1.**
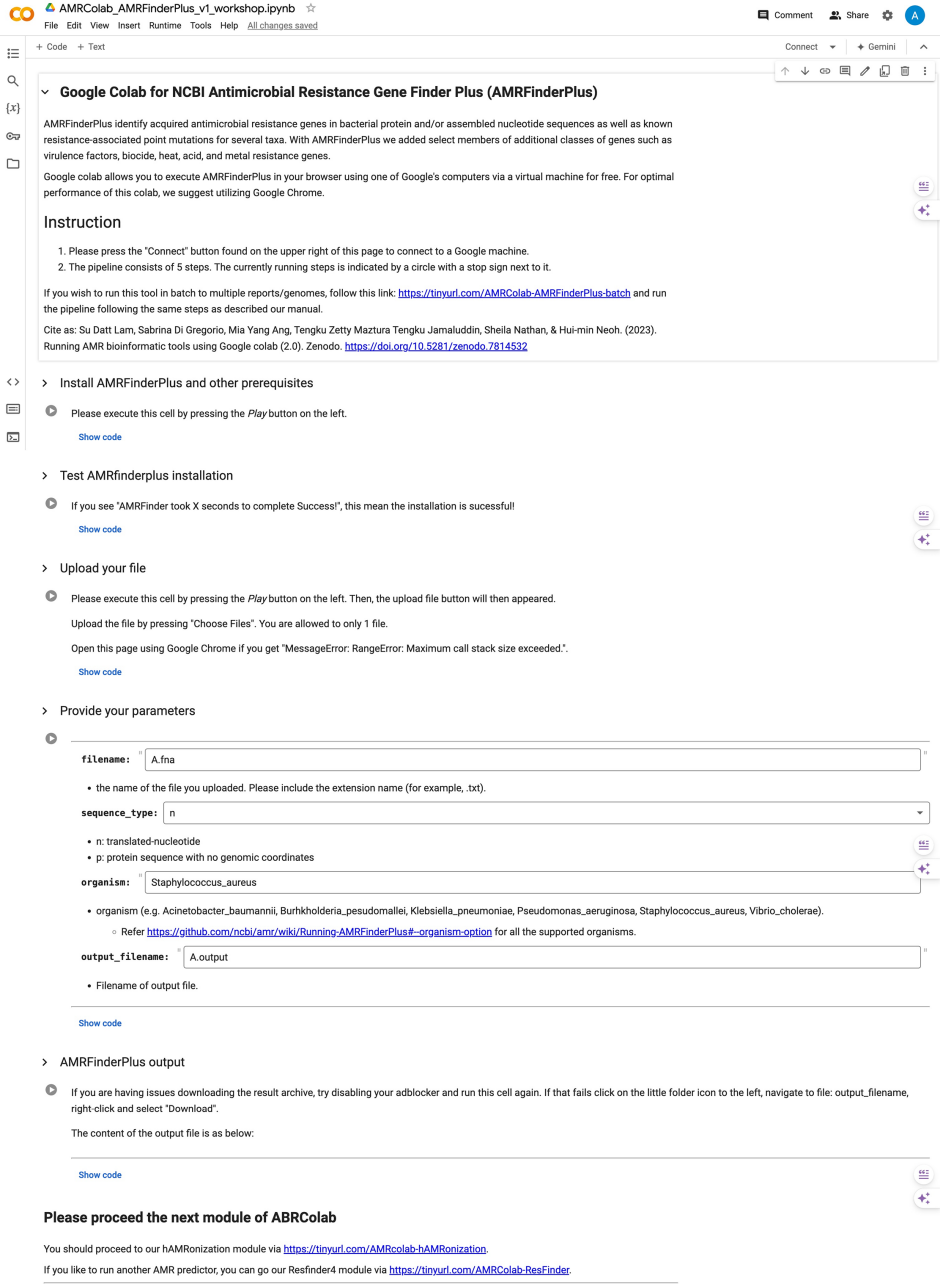
AMRColab AMRFinderPlus module.

To begin using AMRColab, users will key in the URL https://tinyurl.com/AMRColab-AMRFinderPlus into the web browser, where the interface of the Colab will be visible. Step-by-step instructions are available in the interface, prompting users to first connect their computer to the Colab CPU/GPU/TPU, install and test AMRFinderPlus installation, followed by upload of genomic sequences from investigated AMR pathogens (*de novo* assemblies in .fasta file format from any sequencing platform). Users will then key in parameters for their analysis. Once AMR determinants from the genomes via AMRFinderPlus have been identified, the results will be visible under the ‘AMRFinderPlus output’ panel of the Colab. Fig. 1 is a screenshot of the AMRFinderPlus module. For users analysing large quantities of genomes, batch analysis is available via https://tinyurl.com/AMRColab-AMRFinderPlus-batch.

**Fig. 2.**
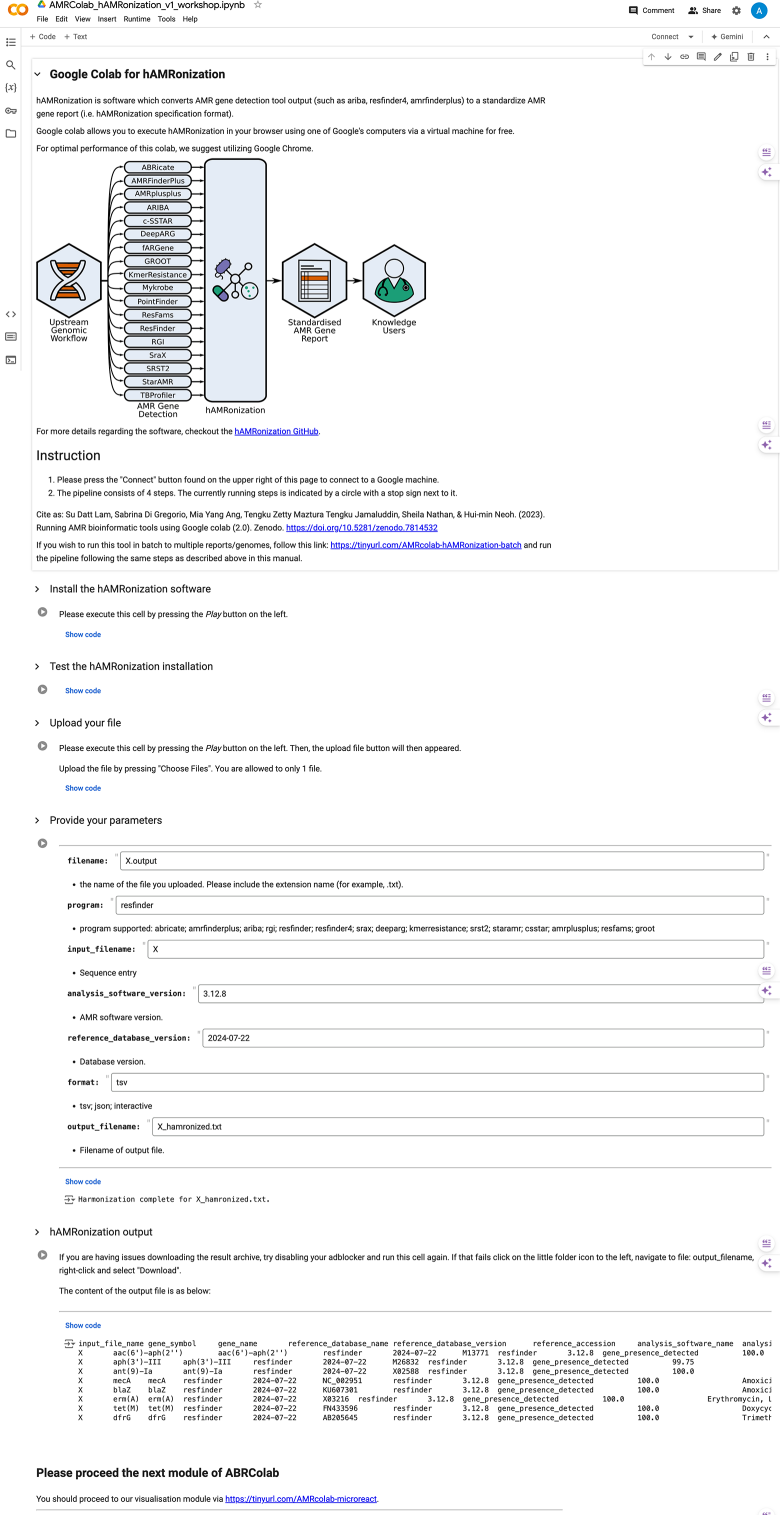
AMRColab hAMRonization module.

Once the AMRFinderPlus analysis is completed by AMRColab, users will be prompted into hAMRonization: https://tinyurl.com/AMRcolab-hAMRonization. Fig. 2 is a screenshot to demonstrate the hAMRonization module. Similar to the previous module, users will be instructed to connect to the Google Colab CPU, install hAMRonization and input analysis parameters. The hAMRonization output will be in the form of a downloadable spreadsheet. Batch analysis of strains is available via https://tinyurl.com/AMRcolab-hAMRonization-batch. In our testing, it took approximately 1 h 32 min to process 200 *S. aureus* genomes.

**Fig. 3.**
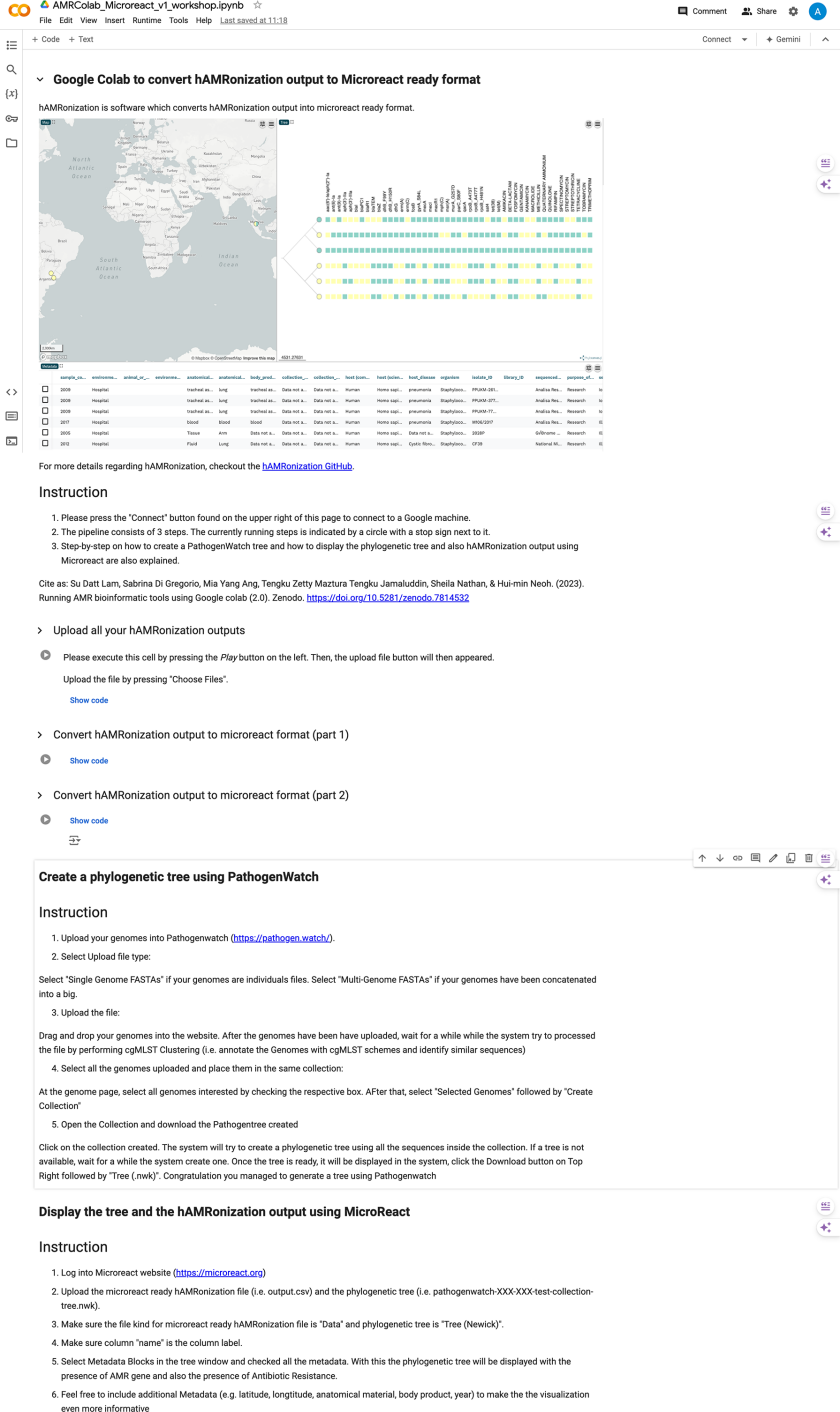
AMRColab visualization module.

Following this, if users choose to visualize their results, they will be directed into the third AMRColab module (https://tinyurl.com/AMRcolab-microreact) containing Pathogenwatch to construct phylogenetic/genomic trees (available only for selected bacterial species) followed by Microreact to produce heat-maps with geospatial locations. Similar to previous modules, users will be prompted step-by-step by instructions available in the Colab. An example of the visualization module and output is shown in Fig. 3.

### AMRColab trial run

Six MRSA strains isolated from Malaysia (PPUKM-261-2009, PPUKM-377-2009, PPUKM-775-2009 and M106_2017) and Argentina (2028P and CF39) were used to test the developed AMRColab. These strains were previously tested and shown to be phenotypically resistant to several antibiotics. PPUKM-261-2009, PPUKM-377-2009 and PPUKM-775-2009 are erythromycin (ERY, macrolide), cefoxitin (FOX, beta-lactam), gentamicin (GEN, aminoglycoside) and penicillin (PEN, beta-lactam) resistant [14]. M106_2017 is only resistant to FOX and PEN [15]. 2028P is PEN, FOX, GEN, ERY, clindamycin (CLIN, lincosamide), ciprofloxacin (CIP, fluoroquinolone), trimethoprim-sulfamethoxazole (TMP, dihydrofolate reductase inhibitor – sulfanilamide) and rifampin (RIF, ansamycin) resistant. CF39 is PEN, FOX, GEN, ERY and tigecycline (TIGE, glycylcycline) resistant [16,17].

**Fig. 4.**
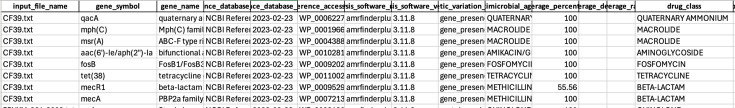
Screenshot of the spreadsheet obtained for tested strains after the first and second modules of AMRColab were executed.

Fig. 4 shows the results obtained after running the first and second modules of AMRColab for the six MRSA strains. The spreadsheet shows information about the tested bacterial strains, their AMR determinants(s) and also the corresponding antibiotic(s) for which the determinants are coding for resistance. The spreadsheet is viewable at: https://tinyurl.com/AMRColab-trial-report.

**Fig. 5.**
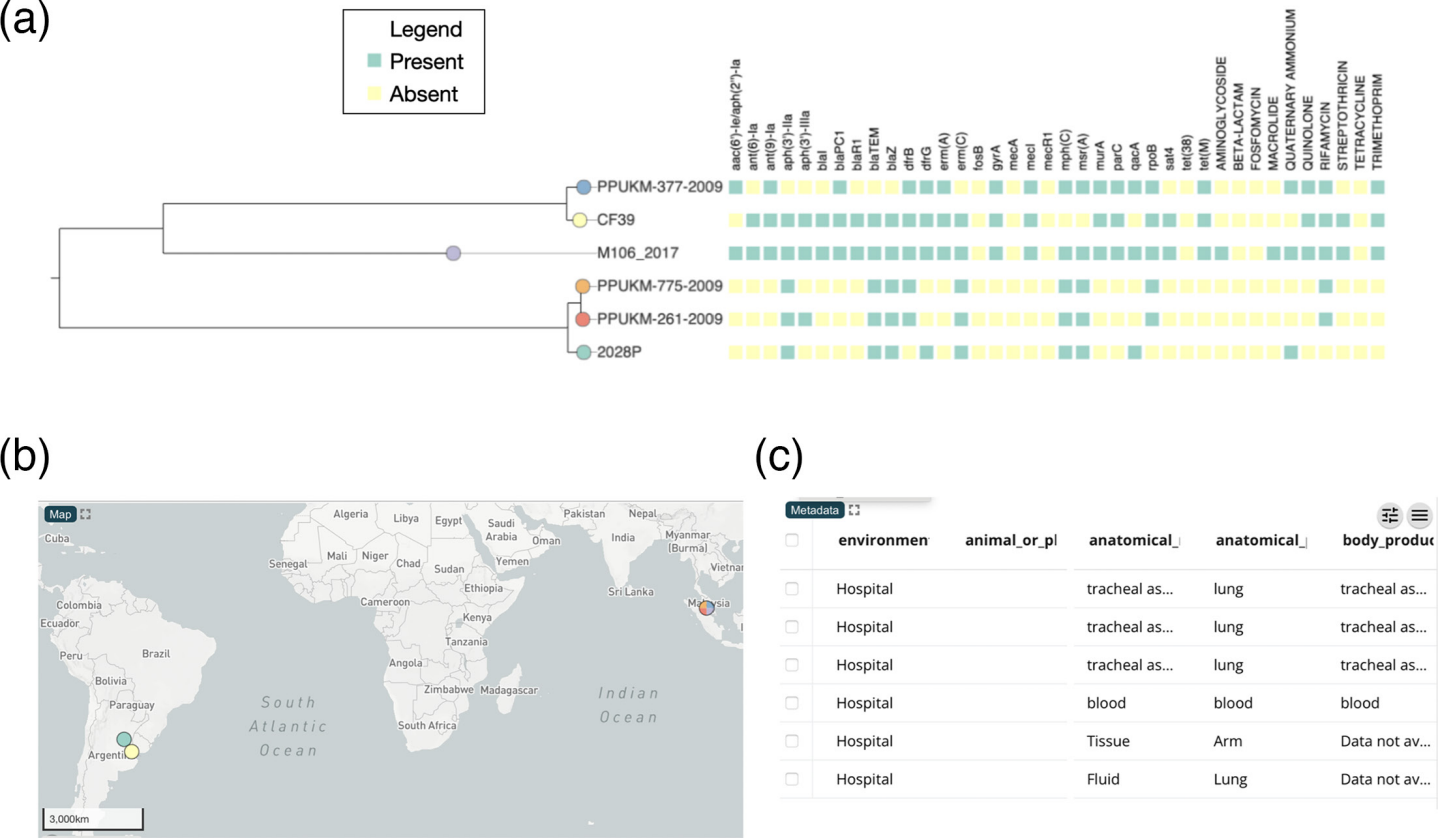
An excerpt of the AMRColab visualization output for tested strains. (**a**) Phylogenetic tree and heatmap of AMR genes. (**b**) Map showing the geospatial locations of the origins of the six strains. (**c**) Additional metadata of the tested strains.

Fig. 5 shows the final visualization output obtained via the third module of AMRColab for the six genomes. All the MRSA genomes carry *fosB*, *mecA* and *tet* genes associated with fosfomycin, beta lactams and tetracycline resistance, respectively. PPUKM-261-2009 and PPUKM-775-2009 belonged to the same clade. They were also found to carry AMR determinants for aminoglycosides, macrolides/lincosamides, quaternary ammonium, fluoroquinolones, streptothricin and trimethoprim. MRSA 2028P, which originated from Argentina, showed a similar AMR determinant profile and clustered closely to PPUKM-261-2009 and PPUKM-775-2009; however, MRSA 2028P did not have quaternary ammonium-resistant determinants but instead harboured an *rpoB* mutation conferring resistance to rifampin. PPUKM-377-2009 formed a clade with CF39 and both strains carried aminoglycoside and macrolide AMR determinants. M106_2017 was a more recent MRSA strain that harboured fewer AMR genes compared to the other tested strains. The output rendered from the visualization module is simple, sleek and suitable for preparing AMR reports, and can be viewed at: https://tinyurl.com/AMRColab-trial-output.

### AMRColab workshop and feedback

Coincidentally, the same number of participants (*n*=32) attended the workshops held in 2022 and 2023. For the workshop held in 2023, most were new attendees; only two participants had previously attended the first workshop held in April 2022 and were attending the second workshop as a refresher course. All participants of the first workshop were staff or graduate students from government-linked Malaysian laboratories or public universities located in the Klang Valley, a central region of Peninsular Malaysia, working on medical microbiology. Approximately 52.0% of the participants were from the Institute for Medical Research (IMR). IMR is an agency under the purview of the Ministry of Health, Malaysia. One of its thrust activities is to release the yearly National Surveillance of Antimicrobial Resistance (NSAR) report for Malaysia. It also serves as the National Focal Point for the WHO’s Collaborative Surveillance Programme on Antibiotic Resistance in the Western Pacific Region and routinely submits national data to the WHO’s Global Antimicrobial Resistance and Use Surveillance System (GLASS). Interestingly, the participants of the second workshop worked in more varied fields of microbiology (medical, food, water, environment, zoonosis, gut microbiota) and were working in laboratories beyond the Klang Valley, such as in East Malaysia (Borneo) (*n*=5, 15.6%), Indonesia (*n*=6, 18.8%), Nepal (*n*=1, 3.1%) and Hungary (*n*=1, 3.1%). For the workshop in 2023, we also collected information about the participants’ experience in bioinformatics. We noted that the majority (*n*=19/32, 67.9%) of them had limited bioinformatics knowledge and a further eight participants (25%) were at the intermediate level.

**Fig. 6.**
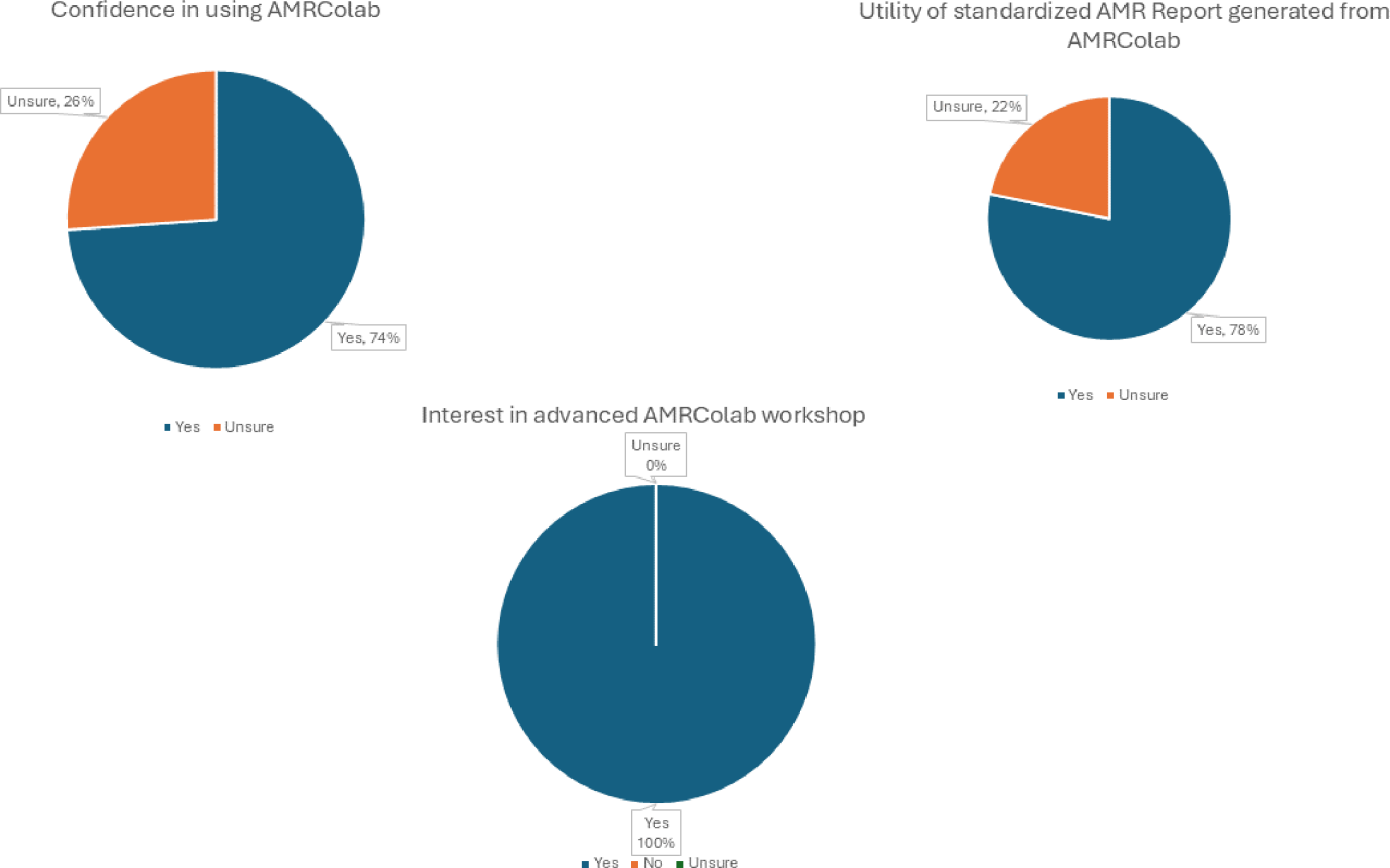
Participant survey responses from the first AMRColab workshop.

A total of 23 and 28 participants, respectively, from the 2022 and 2023 workshops completed and submitted the feedback form (Figs6  7). For the first workshop, 17 respondents (74.0%) felt confident in using AMRColab at the end of the workshop, and 18 (78.0%) of them concurred that the report generated from AMRColab would be useful for their surveillance work (Fig. 6). All respondents stated their interest to participate in future AMRColab workshops. For the second workshop, we noted an improvement in the confidence of the respondents in using AMRColab at the end of the workshop (*n*=26, 92.9%); eight (28.6%) and 13 (46.4%) respondents answered ‘definitely’ and ‘most likely’ to the question of ‘will you be using AMRColab in your research?’. Eleven (39.3%) and 14 (50.0%) respondents gave a score of ‘4’ and ‘5’ (‘5’ being the easiest) about the ease of AMRColab utilization.

**Fig. 7.**
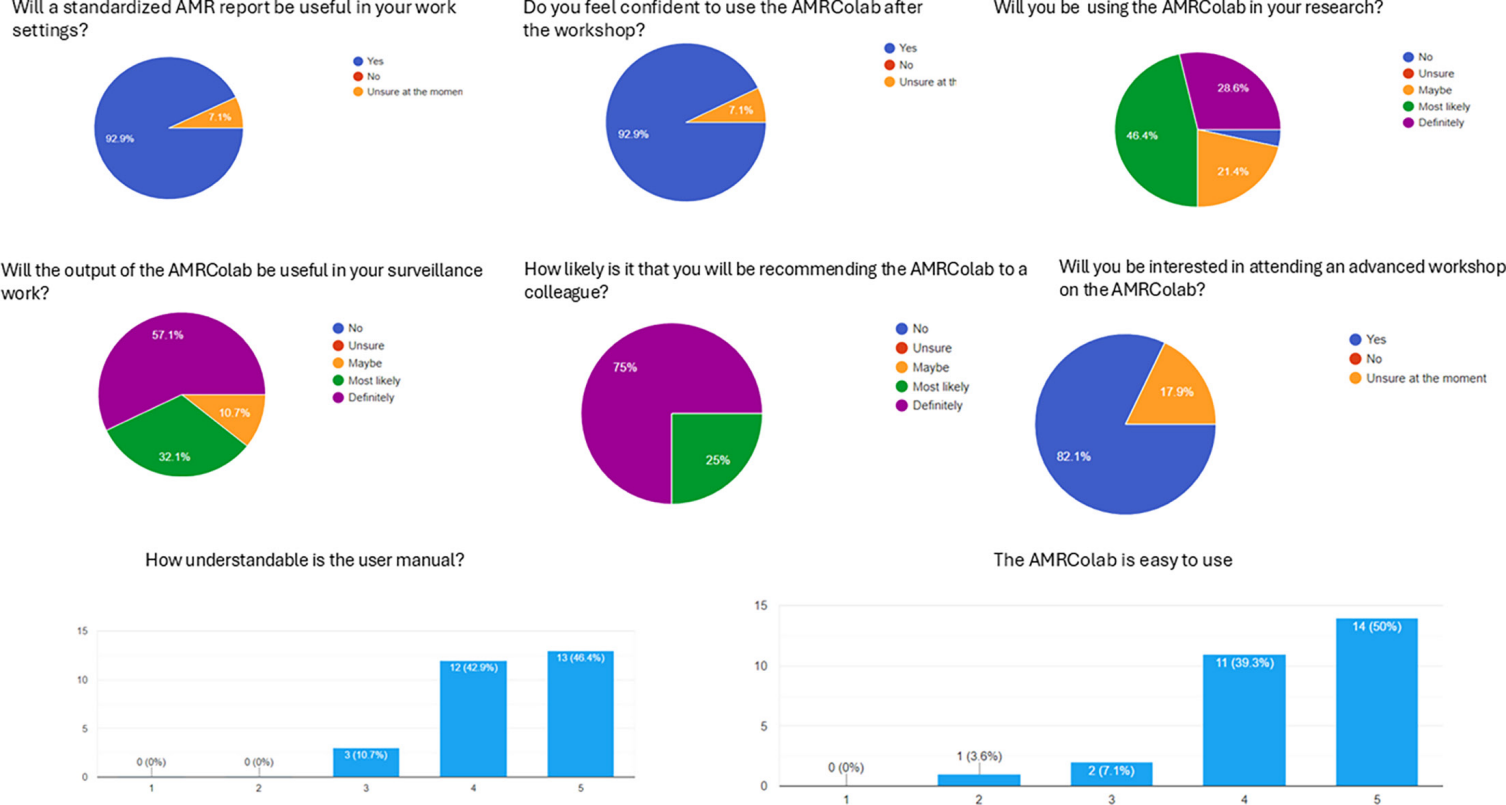
The participants’ survey responses following the second AMRColab workshop and a follow-up survey 12 months after the workshop.

Overall, the feedback from participants of both workshops was positive. They were appreciative that AMRColab could be used by individuals without prior bioinformatics knowledge for the analysis of their AMR pathogens. In the post-workshop utilization survey, ten respondents provided feedback, where six of them (60.0%) had started using AMRColab in their work. Five of these respondents found AMRColab to be useful. The only negative feedback we obtained during the post-workshop utilization survey was from a participant who required the utilization of Resfinder instead of AMRFinderPlus in their analysis. At the time of the workshop, the version of AMRColab had only the AMRFinderPlus analysis pipeline embedded into it, so users who prefer other AMR bioinformatics suites such as ResFinder (http://genepi.food.dtu.dk/resfinder) and ABRicate (https://github.com/tseemann/abricate) would not be able to use AMRColab. We have since added the option of running ResFinder in the Colab (links for ResFinder: https://tinyurl.com/AMRColab-ResFinder and ResFinder batch analysis: https://tinyurl.com/AMRColab-ResFinder-batch) as an alternative to AMRFinderPlus. A participant also mentioned that analyses run in AMRColab could not be saved; however, we do not see this to be problematic as the analysis does not take a long time to complete, and results are in the form of downloadable spreadsheets, with shareable links of visualization maps. No data will be stored in the Google Colab, ensuring data security for the users.

We acknowledge that other tools such as Pathogenwatch also offer a user-friendly analysis of genome assemblies from a web browser without bioinformatics knowledge. However, our approach has the potential to encourage the use of existing bioinformatics tools by public health officers/researchers. AMRColab enables the use of two AMR bioinformatics software (AMRFinderPlus and ResFinder), with a possibility to extend to other software in the future, and to obtain a standardized AMR determinant output (with hAMRonization) that can be compared with those from other bioinformatics tools across public health institutions.

After the workshops, at the time of writing, we have also developed a module for genome assembly (https://tinyurl.com/AMRColab-GenomeAssemblyModule), where users will be able to plug in raw sequence reads from their sequencing platforms for genome assembly prior to the AMR gene identification module. This module uses SPAdes (https://github.com/ablab/spades) for sequence read assembly, followed by QUAST (https://github.com/ablab/quast) for genome quality assessment. The genome assembly and ResFinder modules are currently in beta, and will be introduced and tested in future AMRColab workshops. In our test run, it took 3 h and 35 min to assemble a typical *S. aureus* genome. In addition, among the upcoming improvements to AMRColab include better visualization tools, together with translation of the AMRColab manual into languages other than English. An online community platform for AMRColab users will also be established, together with a tutorial video about the Colab.

## Summary

‘AMRColab’ was developed as an open access AMR bioinformatics analysis suite complete with visualization tools for reporting purposes. Users of AMRColab will be able to easily analyse, compare and visualize trends of circulating AMR pathogen genomes. This would allow timely surveillance of these pathogens by local health authorities, even if these personnel are not trained in bioinformatics, leading towards timely action for prevention of AMR transmission. We envision AMRColab to be a useful tool in AMR laboratories with current limited access to bioinformaticians, breaking down the bioinformatics barrier for easy and timely AMR pathogen genome and epidemiological analysis.

## Abbreviations

AMR: antimicrobial resistance
CIP: ciprofloxacin
CLIN: clindamycin
ERY: erythromycin
FOX: cefoxitin
GEN: gentamicin
GLASS: Global Antimicrobial Resistance and Use Surveillance System
IMR: Institute for Medical Research
MRSA: methicillin-resistant *S*. *aureus*
NCBI: National Center for Biotechnology Information
NSAR: National Surveillance of Antimicrobial Resistance
PEN: penicillin
RIF: rifampin
TIGE: tigecycline
TMP: trimethoprim-sulfamethoxazole
WGS: whole genome sequencing
WHO: World Health Organization
